# *Reticulinasus salahi* (Acarina: Argasidae), a tick of bats and man in the Palaearctic and Afrotropics: review of records with the first pathogens detected

**DOI:** 10.1007/s00436-023-07826-2

**Published:** 2023-04-01

**Authors:** Martin Ševčík, Eva Špitalská, Peter Kabát, Radek K. Lučan, Michaela Maliterná, Antonín Reiter, Marcel Uhrin, Petr Benda

**Affiliations:** 1 Department of Zoology, Faculty of Science, Charles University, Viničná 7, 128 43 Praha 2, Czech Republic; 2 Institute of Virology, Biomedical Research Center, Slovak Academy of Sciences, Dúbravská cesta 9, 845 05 Bratislava, Slovak Republic; 3 Department of Microbiology and Virology, Faculty of Natural Sciences, Comenius University, Ilkovičova 6, 842 15 Bratislava, Slovak Republic; 4 South Moravian Museum in Znojmo, Přemyslovců 129/8, 669 02 Znojmo, Czech Republic; 5 Department of Zoology, Institute of Biology and Ecology, Faculty of Science, P. J. Šafárik University, Šrobárova 2, 041 80 Košice, Slovak Republic; 6 Department of Zoology, National Museum (Natural History), Václavské nám. 68, 115 79 Praha 1, Czech Republic

**Keywords:** *Reticulinasus*, Summary, New records, Mediterranean, Middle East, Pathogens

## Abstract

The soft ticks of the genus *Reticulinasus* Schulze, 1941 (family Argasidae Koch, 1844) are ectoparasites of the fruit bats of the Old World (Pteropodidae). *Reticulinasus salahi* (Hoogstraal, 1953) is the only representative of this genus that occurs in the western part of the Palaearctic. This unusual distribution reflects the distributon range of its primary host, *Rousettus aegyptiacus* (Geoffroy, 1810). In this contribution, we present a revised review of records of this tick that were made in two periods, 1951–1966 (records from Egypt, Israel, Jordan, Spain) and 2005–2019 (Cyprus, Iran, Oman), and additionally, we present notes, re-determinations, new records, and summary of hosts of this tick. Besides the primary host, the revised list of hosts comprises two bats (*Taphozous perforatus* Geoffroy, 1818, *Otonycteris hemprichii* Peters, 1859) and the human (*Homo sapiens* Linnaeus, 1758). We also tried to identify pathogens in specimens of this tick collected from *R. aegyptiacus* in Oman. The DNA of the Mouse herpesvirus strain 68 (MHV-68), of two bacteria, *Borellia burgdorferii* sensu lato, and *Ehrlichia* sp. almost identical (98%) with Candidatus *Ehrlichia shimanensis* was detected in several larvae specimens.

## Introduction

In accordance with a recent revision of the Argasidae ticks based on molecular genetics, the subgenus *Reticulinasus* Schulze, 1941 has to be raised to the genus level (Mans et al. 2021). This genus represents soft ticks parasitizing on the fruit bats (Pteropodidae) of the Old World, and only one species of this genus extends by its distribution to the Palaearctic, the Salah’s Egyptian fruit-bat tampan, *Reticulinasus salahi* (Hoogstraal 1953). This range pattern is a direct consequence of the range extent of its primary host, the Egyptian fruit bat, *Rousettus aegyptiacus* (Geoffroy, 1810); the Palaearctic populations of this bat represent the only geographical offshoot of the fruit bat family (Pteropodidae) out of the tropics.

Records of this tick are rather scarce and most of them were made in the period of 1951–1966 with an additional finding in 2009 (Estrada-Peña et al.^1989^; Hoogstraal 1953; Theodor and Costa 1960; Saliba et al. 1990; Benda et al. 2010). The evidence was reviewed by Sándor et al. (2021), who mapped a distribution range stretching from Spain to the Levant and Egypt. The latter authors also suggested a possible distribution extent of the tick based on the known range of its primary host and added a full list of the recorded hosts besides *R. aegyptiacus*, i.e. *Eptesicus serotinus* (Schreber, 1774), *Taphozopus perforatus* Geoffroy, 1818, and *Homo sapiens* Linnaeus, 1758.

Besides the primary parasitation of the Egyptian fruit bat, *R. salahi* has been evidenced to be a secondary parasite of humans (cf. Hoogstraal 1953; Lavoipierre and Riek 1955); despite this, only marginal attention has been paid to the distribution and ecology of this thick as well as its potential as a vector of pathogens. In the 1950s, only few attempts were made to find spirochaetes and/or salmonellas; however, these surveys failed in finding any of these pathogens (Hoogstraal 1953; Floyd and Hoogstraal 1956). In other bat ticks of the western Palaearctic and Afrotropics, of the genera *Carios* Latreille, 1796 and *Secretargas* Hoogstraal, 1957, the presence of pathogens is enormous. More than twenty species of bacteria and piroplasmids (namely, of the genera *Rickettsia* Da Rocha-Lima, 1916, *Coxiella* Philip, 1948, *Anaplasma* Theiler, 1910 /*Ehrlichia* Moshkovski, 1945, *Bartonella* Strong, Tyzzer, Brues et Sellards, 1915, *Borrelia* Swellengrebel, 1907, and *Babesia* Starcovici, 1893) were reported to be found in two argasid tick species parasitizing bats, which distribution range overlap with the range of *R. salahi* (Sándor et al. 2021).

Taking the aim of a revision of the current status of *R. salahi*, we complemented the review by Sándor et al. (2021) with new and/or revised data from the Middle East collected in 2005–2019. A small part of the newly obtained materials was subjected to a survey of bacteria and piroplasmids of the abovementioned genera. Additionally, a possibility of presence of the Mouse herpesvirus strain 68 (MHV-68), recently confirmed in bats of Europe and Central America (Briestenská et al. 2018; Janíková et al. 2020), was tested.

## Materials and methods

### Study area and materials

The study area covers the distribution range of the Egyptian fruit bat, *Rousettus aegyptiacus*, in the Middle East and north-eastern Africa, at the transition area of two zoogeographic regions, the Palaearctic and Afrotropics. Politically, it comprises wholes or parts of the following countries: Turkey, Cyprus, Syria, Lebanon, Jordan, Egypt, Sudan, Yemen, Oman, United Arab Emirates, and Iran. In these countries, the fruit bats were caught by standard methods, using mist or hand nets. All the body parts of the captured fruit bats (pelage, face, ears, wing membranes) were examined for the parasite presence, and all the ticks found were removed directly in the field by using tweezers and preserved in 96% ethanol. Some additional tick specimens were obtained secondarily, by examinations of the host specimens deposited in the collection of the National Museum, Prague, Czech Republic (NMP). In addition, one record from the Cave at the Sâsân Springat Bishapur, Iran, was realized by a random collection from the cave bottom under the fruit bat colony. Five specimens of *Reticulinasus salahi* were selected for a detailed examination and mounted onto slides using the Swan’s embedding medium (Swan 1936).

### Morphology determination

The adult and nymph ticks specimens stored in alcohol (Table 1) were examined employing standard microscopy (Karl Zeiss Jena, Germany) and compared to published morphology description of the type materials by Hoogstraal (1953: 256–258, Figs. 1–5). Additionally, all larva stages collected in Oman (Table 1) were prepared sufficiently to be identified according to their morphology, and the crucial characters like chelicera, hypostome, palp, Haller’s organ, and dorsal plate (Dumbleton 1959: 307, Text–Fig. 18, Theodor and Costa 1960: 380–381, Text–Fig. 25 a, b, and Sonenshine et al. 1966: 118–120, Figs. 49–50).

**Table 1:** New and corrected published records of *Reticulinasus salahi* arranged by country and date of collection

Country	Locality	Coordinates	Date	Host		Collector/s; host depository†	Number and stage	Detail of collection
Cyprus**	Afendrika	35° 39′ N, 34° 26′ E	17 October 2005	*Rousettus aegyptiacus*	1 ma	leg. R. Lučan	1 f [P]	
Iran*	Bishapur, cave at the Sâsân spring	29° 47′ N, 51° 35′ E	6 October 2011	under colony		leg. A. Reiter	1 n [A]	Benda et al. (2012)
Oman**	Al Hoota cave	23° 06′ N, 57° 22′ E	8 April 2011	*Rousettus aegyptiacus*	1 mj	leg. P. Benda, A. Reiter, M. Uhrin; NMP 93781	5 l [A]^1^	
	Bidbid	23° 25′ N, 58° 08′ E	26 March 2011	*Rousettus aegyptiacus*	1 ma	leg. P. Benda, A. Reiter, M. Uhrin; NMP 93713	2 l [A]	
	Ain Sahnawt	17° 09′ N, 54° 11′ E	27 March 2012	*Rousettus aegyptiacus*	5 ma, 1 mj	leg. P. Benda, A. Reiter, M. Uhrin; NMP 94027–94029	23 l [A, P]^2^	
	Shihayt, Wadi Darbat	17° 09′ N, 54° 28′ E	28 March 2012	*Rousettus aegyptiacus*	2 mj	leg. P. Benda, A. Reiter, M. Uhrin	9 l [A]	
	Wadi Hannah	17° 03′ N, 54° 37′ E	30 March 2012	*Rousettus aegyptiacus*	3 ma, 2 mj, 1 fa, 2 fj	leg. P. Benda, A. Reiter, M. Uhrin; NMP 94064	29 l [A, P]^3^	
	dtto		22 October 2019	*Rousettus aegyptiacus*	1 ma	leg. P. Benda, J. Hájek, A. Reiter; NMP 97051	1 l [A]	

Explanations: *redetermination specimen, first record from country; **new and first record from the country; † the released bats are not mentioned; ^1^ 3 specimens from this collection used in pathogens study; ^2^ 2 specimens from this collection used in pathogens study; ^3^ 1 specimen from this collection used in pathogens study

### Literature sources

All published records of *Reticulinasus salahi* were summarized, starting in 1953 when the species was described (Hoogstraal 1953), till mid-2022 (Table 2). Taxonomy and nomenclature of *Reticulinasus salahi* follow the revision of the family Argasidae by Mans et al. (2021).

**Table 2:** Published records of *Reticulinasus salahi* arranged by country and date of collection

Country	Locality	Coordinates	Date	Host	Sex and age host	Collector and deposited	Detail of collection	References
Spain	Prat de Llobregat (Barcelona)	41° 32′ N, 2° 09′ E	23 June 1955	*Eptesicus serotinus*	ma	leg. F. Lukoschus; not listed	1 l	Estrada-Peña et al. (1989)
Egypt	Hall under Mohammed Ali Mosque, Citadel area, (Cairo)	30° 03′ N, 31° 15′ E	9 May 1951	on the walls		leg. H. Hoogstraal; USNM, No. 2008	m (holotype), f (allotype)	Hoogstraal (1953)
	dtto		various time in 1951–52	on the walls and floors		leg. H. Hoogstraal, A. A. Salah, S. Mittwally, I. S. Khetr, S. Gaber; USNM, FIES, RML, MCZ, FNHM, BMNH, CRBH, DVSO, H. Hoogstraal private collection and other person private collections	400 m, 400 f, 400 n, 80 l (paratypes), 300 laboratory reared larvae from paratype parents	
	Nearby Sultan Hassan Mosque (Cairo)	30° 03′ N, 31° 15′ E		dtto;* Taphozous perforatus*			–; 1 l	
	Fom el Khalig aqueduct in Old Cairo (Cairo)	30° 01′ N, 31° 14′ E		dtto; man [= *Homo sapiens*]			–; “very frequently” records of these ticks “that many engorge”	
	Aquarium grotto, (Cairo)	30° 03′ N, 31° 13′ E		roosts of *Rousettus aegyptiacus*			unknown number l	
	Wadi Natroum, Western Desert	30° 22′ N, 30° 21′ E		dtto			unknown number l	
	Heliopolis (a suburb of Cairo)	30° 06′ N, 31°20′ E		street			1 n	
	The same localities as paratypes					leg. H. Hoogstraal; ZIN AL A853	1 f, 3 m, 1n (paratypes)	Filippova (2008)
	Citadel (Cairo)	30° 03′ N, 31° 15′ E		*Rousettus a. aegypticus *[= *Rousettus aegyptiacus*]			6 l	Sonenshine et al. (1966)
	Gezira Island (Cairo)	30° 03′ N, 31° 13′ E		dtto		leg. H. Hoogstraal; RML 25458	4 l	
Israel	Cave in Herzliah	32° 16′ N, 34° 81′ E	1951	*Rousettus aegyptiacus *		not listed	details of larva were added	Theodor and Costa (1960)
Jordan	Azraq-Shishan	31° 50′ N, 36° 49′ E	2 May 1966	*Myotis* sp. [= *Otonycteris hemprichii*]		leg. S. Atallah; not listed	8 l	Saliba et al. (1990)
Jordan	Iraq Al Amir	31° 55′ N, 35° 45′ E	10 May 2009	under the colony of *R. aegyptiacus*		leg. A. Reiter; CMŠ	4 m, 3 n [A]	Benda et al. (2010)

### Images

**Fig. 1:**
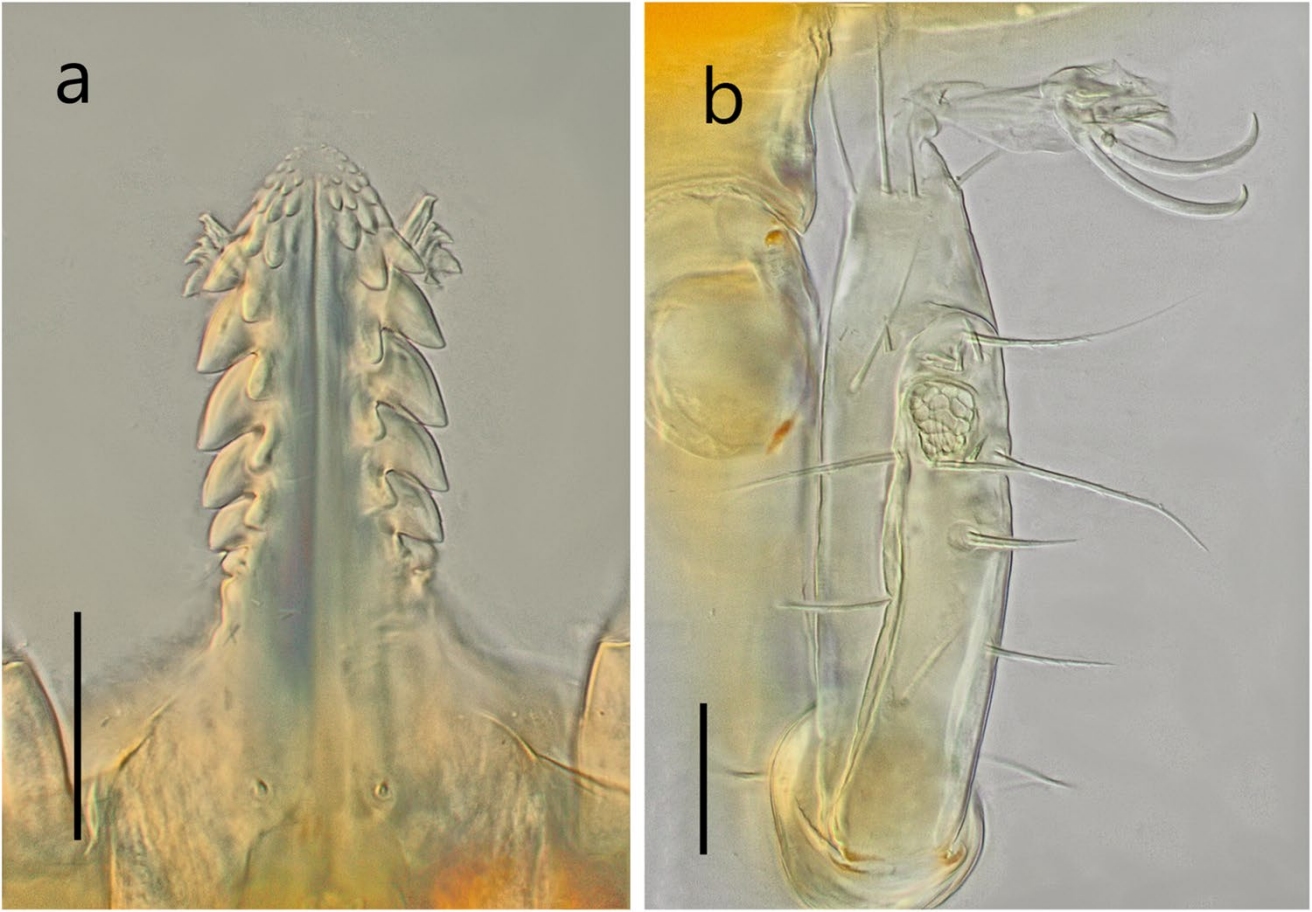
Photomicrograph parts of capitulum of *Reticulinasus salahi* larvae from locality Ain Sahnawt, Oman. **a** Hypostome and partially protruding chelicerae. **b **Terminal segment of the first leg with the Haller’s organ. Scale 50 μm

**Fig. 2:**
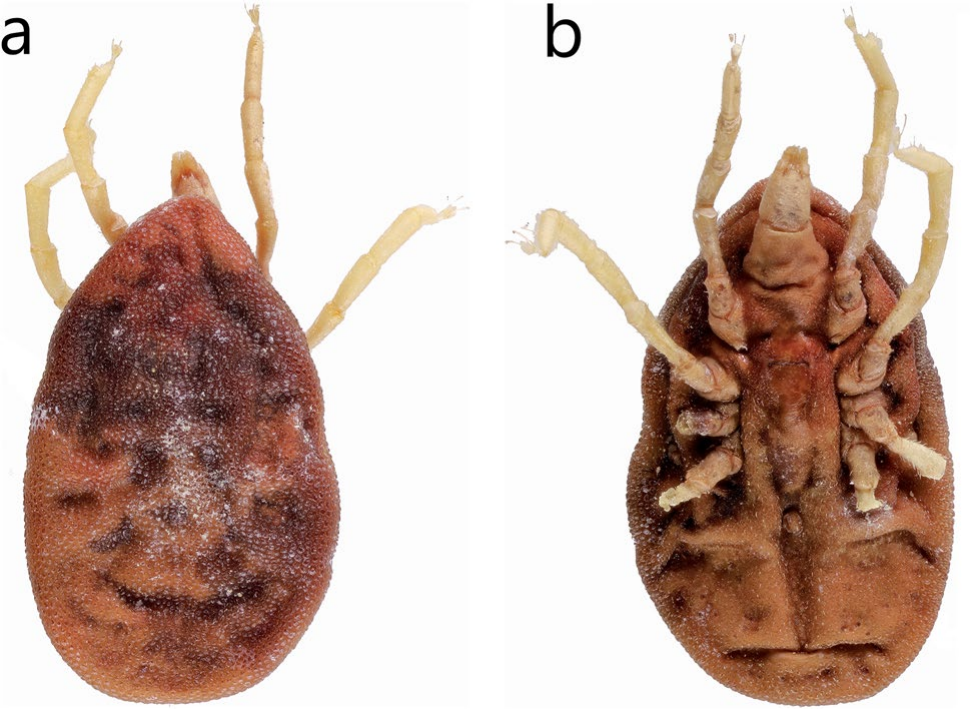
Slightly engorged nymph of *Reticulinasus salahi* from locality Bishapur, cave at the Sâsân spring, Iran, originally identified as *Ornithodoros* sp. in Benda et al. (2012: 530). **a** Dorsal aspect. **b** Ventral aspect

The images of the larvae specimens of *Reticulinasus salahi* from Oman (Fig. 1a, b) were taken by an Olympus XC30 digital camera installed on the Nikon E600 light microscope, where the bright-field and interference contrasts (NomarskyDIC) were applied. For processing the photos, analysis Docu v. 5.1 and Corel Photopaint X5 were employed. The photos of an adult female of *R*. *salahi* from Iran (Fig. 2a, b) were made using a Canon EOS 30D digital camera and Canon MP-E 65/2.8 Macro lens in multiple layers, stacked using Helicon Focus and edited in Corel Photopaint X3.

### Depositories

The list of depositories containing specimens of *Reticulinasus salahi* species is included. The following abbreviations are used: BMNH, Natural History Museum (formerly, British Museum of Natural History), London, UK; FNHM, Field Museum of Natural History (formerly, Chicago Natural History Museum), Chicago, USA; CMŠ, private collection of Martin Ševčík, Nitra, Slovak Republic; CRBH, collection of Dr. R. B. Heisch, Nairobi, Kenya; DVSO, Division of Veterinary Services, Onderstepoort, Pretoria, South Africa; FIES, Fouad I Entomological Society, Cairo, Egypt; MCZ, Museum of Comparative Zoology, Harvard University, Cambridge, USA; NMP, National Museum (Natural History), Prague, Czech Republic; RML, Rocky Mountain Laboratory, Hamilton, MT, USA; USNM, National Museum of Natural History (formerly,United States National Museum), Washington D.C., USA; ZIN, Zoological Institute, Russian Academy of Sciences, St. Petersburg, Russia; others, A, alcoholic preparation; a, adult; f, female; j, juvenil; m, male; l, larva; n, nymph; P, mounted (tick) preparation.

### Pathogen screening and phylogenetic analyses

Selected tick specimens (Table 1) were washed with fresh 70% ethanol, then with sterile water, dried, transferred individually to tubes, and crushed with a sterile Carbon Steel Surgical Scalpel Blade (Surgeon, JAI Surgicals Ltd., India). The DNA from the samples was isolated using QIAamp DNA Mini Kit (Qiagen, Germany) according to the manufacturer’s instructions. The concentration and purity of the DNA were measured by NanoPhotometer Pearl (Implen, Germany). The DNA samples were stored at −20 °C and later used as templates for the PCR amplifications. Tick samples were screened by PCR-based methods for the presence of the MHV-68 virus, bacteria *Rickettsia* spp., *Anaplasma/Ehrlichia* spp., *Borrelia burgdorferi *sensu lato, *Bartonella* spp., and piroplasms *Babesia* spp. (Table 3). The PCR amplicons were purified and analyzed by sequencing in both directions in Macrogen Inc. (Amsterdam, The Netherlands). The DNA sequences were compared with those available in GenBank using the Basic Local Alignment Search Tool (Blast) (http://blast.ncbi.nlm.nih.gov). The new sequence generated in this study was submitted to GenBank under accession number OQ466707.

**Table 3:** Primers used for the detection and/or identification of different vector-borne bacteria in the examined ticks collected from bats

Assay (virus and bacteria)	Primer name	Primer sequence (5′- 3′)	Target gene	A. g. (bp)	A. t. (°C)	Reference
PCR	ORF50 F1	CCACCTGATCAAATATGCCA	ORF50 gene of MHV-68	969	57	Kabát et al. (2021)
MHV–68	ORF50 R1	TGTGGGTTTCTTGTTTGGAC	ORF50 gene of MHV-68			
	ORF50 F2	TGGCATATCCAGAGAAGTTGAG	ORF50 gene of MHV-68	581	57	
	ORF50 R2	TGGGAGTAGGTATGTAGCTCTG	ORF50 gene of MHV-68			
PCR						
*Rickettsia* spp.	SFGF	GAM AAA TGA ATT ATA TAC GCC GCA AA	hypothetical protein (RC0338 gene)	109	60	Socolovschi et al. (2010)
	SFGR	ATT ATT KCC AAA TAT TCG TCC TGT AC				
	SFGP	CTC AAG ATA AGT ATG AGT TAA ATG TAA A				
	RpCs.877p	GGG GGC CTG CTC ACG GCG G	citrate synthase (gltA) gene	380	47	Regnery et al. (1991)
	RpCs.1258n	ATT GCA AAA AGT ACA GTG AAC A				
	Rr190.70p	ATG GCG AAT ATT TCT CCA AAA	outer membrane protein A (ompA) gene	632	54	Roux et al. (1996)
	RR190.701R	GTT CCG TTA ATG GCA GCA TCT				
	17 K-5	GCT TTA CAA AAT TCT AAA AAC CAT ATA	17-kDa antigen gene	434	61	
	17 K-3	TGT CTA TCA ATT CAC AAC TTG CC				
	17kD1	GCT CTT GCA ACT TCT ATG TT		434	61	Anstead and Chilton (2013)
	17kD2	CAT TGT TCG TCA GGT TGG CG				
PCR						
*Anaplasma*/*Ehrlichia* spp.	16S8FE	AGA GTT KGA TCM TGG YTC AG	16rRNA gene spanning the V1 region	470	57	Bekker et al. (2002)
	B-GA1B	CGA GTT TGC CGG GAC TTY TTC T	16rRNA gene spanning the V1 region			
PCR						
*Borrelia burgdorferi* sensu lato	Bb23Sf	CGAGTCTTAAAAGGGCGATTTAGT	23S rRNA	77	60	Courtney et al. (2004)
	Bb23Sr	GCTTCAGCCTGGCCATAAATAG				
	Bb23Sp	6-FAM-AGATGTGGTAGACCCGAAGCCGAGTG-TAMRA				
	IGSa	CGA CCT TCT TCG CCT TAA AGC	rrfA-rrlB intergenic spacer (ITS)	225–255	56	Derdáková et al. (2003)
	IGSb	AGC TCT TAT TCG CTG ATG GTA-3				
PCR						
*Bartonella* spp.	BA325s	CTT CAG ATG ATG ATC CCA AGC CTT CTG GCG	16S–23S rRNA gene ITS region	420–780	66	Maggi et al. (2009)
	BA1100as	GAA CCG ACG ACC CCC TGC TTG CAA AGC A	16S–23S rRNA gene ITS region			
PCR						
*Babesia* spp.	BJ1	GTC TTG TAA TTG GAA TGA TGG	18S rRNA	450	55	Casati et al. (2006)
	BN2	TAG TTT ATG GTT AGG ACT ACG	18S rRNA			

## Results

### Comments on records

Hoogstraal (1953: 256) reported the first record of *Reticulinasus salahi* from Israel; he mentioned the specimens collected in Jerusalem by O. Theodor. An additional locality of this tick from Israel was mentioned by Theodor and Costa (1960: 381), who found it on *Rousettus aegyptiacus* “in a cave in Herzliah together with specimens of *Ornithodorus tholozani* in 1951.” However, Theodor and Costa (1960: 381) also commented Hoogstraal’s (1953) report as follows: “Hoogstraal, in a footnote in his paper on *O*. *salahi* [= *Reticulinasus salahi*] mentions that it has been found in Jerusalem. This is not correct, the only locality in which it has been found so far is Herzliah in the coastal plain. This mistake has been taken over by Leeson (1955 [= 1956]) in his second paper on the distribution of species of *Ornithodorus*.”

A similar situation appeared, when Hoogstraal (1962: 185) discussed the distribution of the genus *Reticulinasus* in Lebanon as follows: “Members of this subgenus are […] [*Ornithodoros* (*Reticulinasus*)] *salahi* Hoogstraal of Egypt, Lebanon, and Palestine.” Nevertheless, we did not find any record of this tick from Lebanon (for a review, see Benda et al. 2016). Thus, we are unsure whether this note refers to an unpublished record or represents just an assumption based on the range of its primary host, *R. aegyptiacus*, in the Middle East.

Benda et al. (2012: 530) reported a single record of *Ornithodoros* sp. from Iran originally published with the following note: “An adult female of the tick *Ornithodoros* sp. was sampled from the bottom of the cave at the Sasan spring at Bishapur (Fars) where colonies of *Rousettus aegyptiacus*, *Rhinopoma microphyllum*, *R*. *muscatellum*, *Myotis blythii*, and *Miniopterus pallidus* were found.” We examined the specimen in detail and clearly identified it as a nymph of *Reticulinasus salahi* (Fig. 2a, b).

On the other hand, we regard the reported finding of *R. salahi* from *Eptesicus serotinus* in Prat de Llobregat (Barcelona), Spain (Estrada-Peña et al. 1989, also in Cordero del Campillo et al. 1994), as doubtful, requiring a revision. The authors mentioned the deposition of the specimen at the Parasitology Unit of the Faculty of Veterinary Medicine of Zaragoza; however, we were not able to obtain it for a revision. Regarding the geographic distance to other record sites and the reported host species, we consider the species identification of the tick as erroneous (at least temporarily), until other records supporting such geographically and ecologically extraordinary findings are available. Estrada-Peña et al. (1989) considered this record unusual and accidental. In the western part of the Mediterranean, the bat species of the genus *Eptesicus* are primary hosts of other tick species, *Secretargas transgariepinus* (Beaucournu and Clerc 1968; Médard et al. 1997; own unpublished data from Morocco).

Finally, Saliba et al. (1990: 164) reported a record of *Reticulinasus salahi* (as *Ornithodoros salahi*) from Jordan being found on “*Myotis* sp., […] Azraq-Shishan, 2.v.1966.” However, the bat specimens originally identified as *Myotis* sp., collected in May 1966 in Azraq-Shishan, represent in fact *Otonycteris hemprichii* Peters, 1859 (see Atallah (1967) and Benda et al. (2010)).

Newly collected specimens of *Reticulinasus salahi* were found on *Rousettus aegyptiacus* examined in two countries of the Middle East, Cyprus, and Oman (Table 1, Fig. 1a, b).

The morphologic characters by Dumbleton (1959) and Sonenshine et al. (1966) mentioned as additional for description and identification of the larvae of *R*. *salahi*, i.e., the shape of dorsal plate and/or capsule of Haller’s organ, are very variable in the Omani specimens (Fig. 1b).

### Pathogens

DNA samples were screened for the presence of MHV-68 virus by nested PCR targeting the ORF50. Of the six analyzed samples (3 larvae from Al Hoota cave, 2 larvae from Ain Sahnawt and 1 larva from Wadi Hannah, Oman; Table 1) of the genomic DNA isolated from the larvae of *Reticulinasus salahi*, the presence of the ORF50 sequence of the MHV-68 virus was found in one sample (Al Hotta Cave, Oman). The obtained sequence of the ORF 50 nested PCR fragment showed 100% homology (position from 68,219 to 68,799 nucleotides) only with the major lytic transactivator protein, which is specific for this virus, and 85% homology with wood mouse herpesvirus. In addition, the temperature profile of the PCR reaction was designed in such a way that amplification of partially homologous sequences does not occur. The presence of the MHV-68 strain has been documented for the first time in this part of the Middle East.

On the other hand, all the analyzed samples of *Reticulinasus salahi* larvae were negative for the presence of the DNA of *Rickettsia*, *Bartonella*, and *Babesia* spp. One tick sample (Al Hotta Cave, Oman) was positive for *Borellia burgdorferi* by real-time PCR. However, since the ct value was > 36, it was not successfully sequenced. The DNA extract of the *R. salahi* larva collected from *Rousettus aegyptiacus* in Wadi Hannah (Oman) was PCR positive for the presence of the *Anaplasma*/*Ehrlichia* 16S rRNA. In this sample, the sequences (GeneBank Accession Number OQ466707) were identical for 97.8% to the Candidatus *Ehrlichia shimanensis* (GeneBank Accession Number AB074459).

## Discussion

**Fig. 3:**
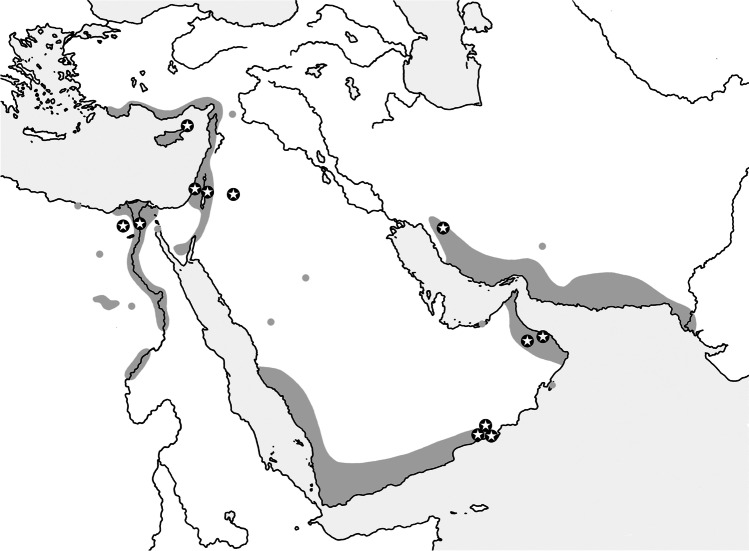
Map of the records of *Reticulinasus salahi* (star dots) against the Palaearctic distribution range of its principal host, *Rousettus aegyptiacus* (dark gray areas); the gray dots denote the records of *R. aegyptiacus* out of its continuous range. For the parasite records, see text and Tables 1 and 2; the host range is reconstructed after Benda et al. (2011, 2012), Judas et al. (2018), and Benda and Ševčík (2020). The alleged record of *R. salahi* from Spain is not depicted (see text for details)

Specimens of the tick *Reticulinasus salahi* were collected in two separate periods. In 1951–1966, the species was described and the first data on its natural history were gathered. Several species of hosts were documented at that time: *Rousettus aegyptiacus*, *Eptesicus serotinus*, *Taphozous perforatus*, *Myotis* sp. [= *Otonycteris hemprichii*], and *Homo sapiens*. The records were made mainly in Egypt and additionally also in Israel, Jordan, and Spain (Hoogstraal 1953; Theodor and Costa 1960; Estrada-Peña et al. 1989; Saliba et al. 1990; Benda et al. 2010). In 2005–2019, new records are available only from one host species, *Rousettus aegyptiacus*. The latter records findings come from specialized trips organized to investigate the bat fauna of the Middle East (Benda et al. 2007, 2010, 2012) (Fig. 3). However, the records of *R. salahi* from the northern Levant (Israel and Lebanon) remain uncertain. On the other hand, we identified a finding from Iran, which represents new extension of the species distribution range. The westernmost record of *R. salahi*, reported from *Eptesicus serotinus* collected in Spain (Estrada-Peña et al. 1989), does not conform to other records considering the host species as well as the distribution area. We suggest to consider it as dubious until it is revised and the species identification confirmed without doubts.

Collections of ticks in free habitats (off the hosts) and checks the tick presence in various habitats still miss, despite the records made in recent time. Considering the primary host range, the available records of *R. salahi* come from just a fragment of the expected distribution range. Based on his personal records, Hoogstraal (1953) regarded *R*. *salahi* to be by far the most common tick parasite of bats in the downtown of Cairo; its density and abundance thus could be very high. A factor influencing the occurrence of *R. salahi* could be the size colony of the Egyptian fruit bats, the primary host. Already, Hoogstraal (1953: 260) reported that in the course of 2 years, he searched for this tick in tens of potential roosts (caves and artificial spaces) in the Cairo region and surrounding areas of Lower Egypt, but he found of *R. salahi* only in the proper area of Cairo in three sites (Mohammed Ali Mosque in the Citadel, Sultan Hassan Mosque, Fom el Khalig). In all cases, the sites of findings were roosts of very large colonies of *R. aegyptiacus*. Another important factor influencing the obvious presence of ticks in the bat roost could be the day period; Hoogstraal (1953: 261) noted as follows: “Engorged larvae can easily be found among moist bat droppings on the floor at each site where fruit bats rest. Nymphs and adults rest among bat droppings, under rocks, or in lower wall crevices. They commence crawling upwards on walls toward midday.” Benda et al. (2011) summarized the records of colonies *R. aegyptiacus* throughout its Palaearctic range; this review could be used for searching of the tick occurrence.

The evidence of the MHV-68 virus in specimens from Oman (Al Hoota Cave) includes *R*. *salahi* among possible vectors of this pathogen among ticks; the DNA of this virus was detected already in the ixodid species *Dermacentor reticulinasus* and *Ixodes ricinus* (Kúdelová et al. 2015, 2017, 2018). Besides ticks, one of the main reservoirs of this virus is rodents (Blaškovič et al. 1980; Mistríková and Blaskovic 1985; Hricová and Mistríková 2008). However, according to the results of laboratory experiments, *R*. *salahi* does not parasitize other vertebrates (Lavoipierre and Riek 1955), and this fact suggests that another reservoir of the MHV-68 virus could be bats and/or humans (primates), the only two known groups of hosts of *R. salahi*. In both groups of hosts, this virus was already confirmed (Briestenská et al. 2018; Janíková et al. 2020; Wágnerová et al. 2015). Our new finding of this virus in Oman represents the southernmost known occurrence spot of this pathogen in the Old World. Our results also support the hypothesis that the MHV-68 virus is a globally widespread herpesvirus capable of inter-species transmission, using one of the suitable vectors available on the site. Now is clear that *R. salahi* is another tick species which could serve as a reservoir of the virus and play a certain role in its ecology and epidemiology.

One larva of *R. salahi* collected in Oman (Al Hotta Cave) was positive for *Borellia burgdorferi* s.l. by real-time PCR. Among the soft ticks parasitizing bats, the presence of *B. burgdorferii* s.l. was documented only in *Carios vespertilionis* Latreille, 1796 (Hubbard et al. 1998). This tick species was collected from *Rousettus aegyptiacus* at Lokwi in South Sudan (Hoogstraal 1956), so the parasitation of this bat by *C. vespertilionis* cannot be excluded also in other parts of its range, including the Palaearctic—this tick is a common parasite of the vespertilionid bats in the latter region (Sándor et al. 2021).

The presence of the Candidatus *Ehrlichia shimanensis* DNA was discovered in a larva of *R*. *salahi* from *Rousettus aegyptiacus* collected at Wadi Hannah in Oman. The Candidatus *E. shimanensis* has been known only from the temperate zone of Central and East Asia, found in game species and small rodents, and also in the hard tick *Haemaphysalis longicornis* Neumann, 1901 (Kawahara et al. 2006; Rar et al. 2008). The vectors of *Ehrlichia* sp. are hard ticks and any connection with the soft ticks has been unknown (Socolovschi et al. 2012). Further studies are needed to describe in detail these agents and determined whether *R*. *salahi* could really represent their vector and/or reservoir.

## Data Availability

The sequences obtained in this study are deposited in GenBank. All other relevant data are included in the manuscript and the references are available upon request from the corresponding author.

## References

[CR1] Anstead CA, Chilton NB (2013). A novel *Rickettsia* species detected in vole ticks (*Ixodes*
*angustus*) from western Canada. Appl Environ Microbiol.

[CR2] Atallah SI (1967) Mammalogy (with a list of reptiles and amphibians). In: Boyd JE (ed) International Jordan Expedition 1966. Unpublished report. London: International Biological Programme, Conservation of Terrestrial Communities Section, 56–63

[CR3] Beaucournu JC, Clerc B (1968) Documents faunistiques et écologiques *Argas *(*Sectretargas*) *transgariepinus* White, 1846, tique nouvelle pour la France et l’Algerie. Vie et Milieu, Obser Océanol– Labor Arago 1968:233–236

[CR4] Bekker CPJ, de Vos S, Taoufik A, Sparagano OAE, Jongejan F (2002). Simultaneous detection of *Anaplasma* and *Ehrlichia* spp. in ruminants and detection of *Ehrlichia*
*ruminantium* in *Amblyomma*
*variegatum* ticks by reverse line blot hybridization. Vet Microbiol.

[CR5] Benda P, Ševčík M (2020). Bats (Mammalia: Chiroptera) of the Eastern Mediterranean and Middle East. Part 16. Review of the distribution and taxonomy of bats in Egypt. Acta Soc Zool Bohem.

[CR6] Benda P, Hanák V, Horáček I, Hulva P, Lučan R, Ruedi M (2007) Bats (Mammalia: Chiroptera) of the Eastern Mediterranean. Part 5. Bat fauna of Cyprus: review of records with confirmation of six species new for the island and description of a new subspecies. Acta Soc Zool Bohem 71:71–130

[CR7] Benda P, Lučan RK, Obuch J, Reiter A, Andreas M, Bačkor P, Bohnenstengel T, Eid EK, Ševčík M, Vallo P, Amr ZS (2010). Bats (Mammalia: Chiroptera) of the Eastern Mediterranean and Middle East. Part 8. Bats of Jordan: fauna, ecology, echolocation, ectoparasites. Acta Soc Zool Bohem.

[CR8] Benda P, Abi-Said M, Bartonička T, Bilgin R, Faizolahi K, Lučan RK, Nicolaou H, Reiter A, Shohdi W, Uhrin M, Horáček I (2011). *Rousettus*
*aegyptiacus* (Pteropodidae) in the Palaearctic: list of records and revision of the distribution range. Vespertilio.

[CR9] Benda P, Faizolâhi K, Andreas M, Obuch J, Reiter A, Ševčík M, Uhrin M, Vallo P, Ashrafi S (2012). Bats (Mammalia: Chiroptera) of the Eastern Mediterranean and Middle East. Part 10. Bat fauna of Iran. Acta Soc Zool Bohem.

[CR10] Benda P, Abi Said MR, Bou Jaoude I, Karanouh R, Lučan RK, Sadek R, Ševčík M, Uhrin M, Horáček I (2016). Bats (Mammalia: Chiroptera) of the Eastern Mediterranean and Middle East. Part 13. Review of distribution and ectoparasites of bats in Lebanon. Acta Soc Zool Bohem.

[CR11] Blaškovič D, Stančeková M, Svobodová J, Mistríková J (1980). Isolation of five strains of herpesviruses from two species of free living small rodents. Acta Virol.

[CR12] Briestenská K, Janíková M, Kabát P, Csepányiová D, Zukal J, Pikula J, Kováčová V, Linhart P, Banďouchová H, Mistríková J (2018). Bats as another potential source of murine gammaherpesvirus 68 (MHV-68) in nature. Acta Virol.

[CR13] Casati S, Sager H, Gern L, Piffaretti J-C (2006). Presence of potentially pathogenic *Babesia* sp. for human in *Ixodes*
*ricinus* in Switzerland. Ann Agric Environ Med.

[CR14] Cordero del Campillo M, Castañón Ordóñez L, Reguera Feo A (1994) Índice Catálogo de Zooparásitos Ibéricos. 2ª Edición. Secretariado de Publicaciones, Universidad de León, León

[CR15] Courtney JW, Kostelnik LM, Zeidner NS, Massung RF (2004). Multiplex real-time PCR for detection of *Anaplasma*
*phagocytophilum* and *Borrelia*
*burgdorferi*. J Clin Microbiol.

[CR16] Derdáková M, Beati L, Peťko B, Stanko M, Fish D (2003). Genetic variability within *Borrelia*
*burgdorferi* sensu lato genospecies established by PCR-single-strand conformation polymorphism analysis of the rrfA-rrlB intergenic spacer in *Ixodes*
*ricinus* ticks from the Czech Republic. Appl Environ Microbiol.

[CR17] Dumbleton LJ (1959) Bat-infesting *Ornithodoros* (Ixodoidea-Argasidae) of the Oriental-Australian region. Proc Linn Soc New South Wales 83:303–308

[CR18] Estrada-Peña A, Sánchez Acedo C, Peribáñez López MA (1989). Nuevos datos relativos a la distribución de los Ixodoidea en España (IV): Primera cita de *Ornithodoros* (*Reticulinasus*) *salahi* Hoogstraal, 1953 (Acarina: Argasidae). Rev Ibér Parasitol.

[CR19] Filippova NA (2008). Type specimens of argasid and ixodid ticks (Ixodoidea: Argasidae, Ixodidae) in the collection of the Zoological Institute, Russian Academy of Sciences (St. Petersburg). Entomol Rev.

[CR20] Floyd TM, Hoogstraal H (1956). Isolation of *Salmonella* from ticks in Egypt. J Egypt Pub Health Ass.

[CR21] Hoogstraal H (1953). *Ornithodoros*
*salahi* sp. nov. (Ixodoidea, Argasidae) from the Cairo Citadel, with notes on *O*. *piriformis* Warburton, 1918 and *O*. *batuensis* Hirst, 1929. J Parasitol.

[CR22] Hoogstraal H (1962). Description of *Ornithodoros* (*Reticulinasus*) *madagascariensis* n. sp. (Ixodoidea, Argasidae). Acarologia.

[CR23] Hoogstraal H (1956) African Ixodoidea. I. Ticks of the Sudan (with Special Reference to Equatoria Province and with preliminary reviews of the genera *Boophilus*, *Margaropus*, and *Hyalomma*). Department of the Navy, Washington

[CR24] Hricová M, Mistríková J (2008). Ecological characterization of murine gammaherpesvirus 68 and its epidemiological implications. Biologia Bratislava.

[CR25] Hubbard MJ, Baker AS, Cann KJ (1998). Distribution of Borrelia burgdorferi sl spirochaete DNA in British ticks (Argasidae and Ixodidae) since the 19th century, assessed by PCR. Med Vet Entomol.

[CR26] Janíková M, Briestenská K, Salinas-Ramos VB, Mistríková J, Kabát P (2020). Molecular detection of murine gammaherpesvirus 68 (MHV-68) in bats from Mexico. Acta Virol.

[CR27] Judas J, Csorba G, Benda P (2018). The bat fauna (Mammalia: Chiroptera) of the United Arab Emirates: a review of published records and museum specimens with conservation notes. J Threat Taxa.

[CR28] Kabát P, Briestenská K, Ivančová M, Trnka A, Špitalská E, Mistríková J (2021). Birds belonging to the family Paridae as another potential reservoir of murine gammaherpesvirus 68. Vect Born Zoon Dis.

[CR29] Kawahara M, Rikihisa Y, Lin Q, Isogai E, Tahara K, Itagaki A, Hiramitsu Y, Tajima T (2006). Novel genetic variants of *Anaplasma*
*phagocytophilum*, *Anaplasma*
*bovis*, *Anaplasma*
*centrale*, and a novel *Ehrlichia* sp. in wild deer and ticks on two major islands in Japan. Appl Environ Microbiol.

[CR30] Kúdelová M, Belvončíková P, Vrbová M, Kovaľová A, Štibrániová I, Kocáková P, Slovák M, Špitalská E, Lapuníková B, Matúšková R, Šupolíková M (2015). Detection of MHV-68 in *Dermacentor*
*reticulatus* ticks. Microb Ecol.

[CR31] Kúdelová M, Jánošová M, Vrbová M, Matúšková R, Slovák M, Belvončíková P (2017). Detection of transcripts and an infectious dose of murine gammaherpesvirus 68 in *Dermacentor*
*reticulatus* ticks. J Infect Dis Ther.

[CR32] Kúdelová M, Jánošová M, Belvončíková P (2018). First detection of murine herpesvirus 68 in adult *Ixodes*
*ricinus* ticks. Folia Microbiol.

[CR33] Lavoipierre MMJ, Riek RF (1955). Observations on the feeding habits of argasid ticks and on the effect of their bites on laboratory animals, together with a note on the production of coxal fluid by several of the species studies. Ann Trop Med Parasitol.

[CR34] Leeson H (1956). Further notes on the geographical distribution of Old World species of *Ornithodoros* (Acarina). Bull Entomol Res.

[CR35] Maggi RG, Kosoy M, Mintzer M, Breitschwerdt EB (2009). Isolation of candidatus *Bartonella*
*melophagi* from human blood. Emerg Infect Dis.

[CR36] Mans BJ, Kelava S, Pienaar R, Featherston J, de Castro MH, Quetglas J, Reewes WK, Durden LA, Miller MM, Laverty TM, Shao R, Takano A, Kawabata H, Moustafa MAM, Nakao R, Matsuno K, Greay TL, Evasco KL, Barker D, Barker SC (2021) Nuclear (18S-28S rRNA) and mitochondrial genome markers of *Carios* (*Carios*) *vespertilionis* (Argasidae) support *Carios* Latreille, 1796 as a lineage embedded in the Ornithodorinae: re-classification of the *Carios* sensu Klompen and Oliver (1993) clade into its respective subgenera. Ticks Tick-Borne Dis 12:101688. 10.1016/j.ttbdis.2021.101688 10.1016/j.ttbdis.2021.101688 33652332

[CR37] Médard P, Guiguen C, Beaucournu JC (1997). Nouvelles récoltes d’*Argas*
*transgariepinus* White, 1846 tique de Chiroptères (Acarina – Ixodoidea – Argasidae) en France et au Maroc. Bull Inf Pathol Anim Sauv.

[CR38] Mistríková J, Blaskovic D (1985). Ecology of the murine alphaherpesvirus and its isolation from lungs of rodents in cell culture. Acta Virol.

[CR39] Rar VA, Livanova NN, Panov VV, Kozlova IV, Pukhovskaya NM, Vysochina NP, Tkachev SE, Ivanov LI (2008) Prevalence of *Anaplasma* and *Ehrlichia* species in Ixodes persulcatus ticks and small mammals from different regions of the Asian part of Russia. Int J Med Microbiol 298:222–230. 10.1016/j.ijmm.2008.01.001

[CR40] Regnery RL, Spruill CL, Plikaytis BD (1991). Genotypic identification of rickettsiae and estimation of intraspecies sequence divergence for portions of two rickettsial genes. J Bacteriol.

[CR41] Roux V, Fournier PE, Raoult D (1996). Differentiation of spotted fever group rickettsiae by sequencing and analysis of restriction fragment length polymorphism of PCR-amplified DNA of the gene encoding the protein rOmpA. J Clin Microbiol.

[CR42] Saliba EK, Amr ZS, Wassef HY, Hoogstraal H, Main AJ (1990). The ticks (Ixodoidea) of East Jordan and the West Bank. Dirasat S B.

[CR43] Sándor AD, Mihalca AD, Domşa C, Péter Á, Hornok S (2021). Argasid ticks of Palearctic bats: distribution, host selection, and zoonotic importance. Front Vet Sci.

[CR44] Socolovschi C, Mediannikov O, Sokhna C, Tall A, Diatta G, Bassene H, Trape JF, Raoult D (2010) *Rickettsia felis*-associated Uneruptive Fever, Senegal. Emerg Infect Dis 16:1140–1142. 10.3201/eid1607.100070 10.3201/eid1607.100070 PMC3321914 20587190

[CR45] Socolovschi C, Kernif T, Raoult D, Parola P (2012) *Borrelia*, *Rickettsia*, and *Ehrlichia *species in bat ticks, France, 2010. Emer Infect Dis 18(12):1966–1975. 10.3201/eid1812.111237 10.3201/eid1812.111237 PMC3557878 23171714

[CR46] Sonenshine DE, Clifford CM, Kohls GM (1966). The systematics of the subfamily Ornithodorinae (Acarina: Argasidae). III. Identification of the larvae of the Eastern Hemisphere. Ann Entomol Soc America.

[CR47] Swan DC (1936) Berlese’s fluid: remarks upon its preparation anduse as a mounting medium. Bull Ent Res 27:389–391. 10.1017/S0007485300058259

[CR48] Theodor O, Costa M (1960). New species and new records of Argasidae from Israel. Observations on the rudimentary scutum and the respiratory system of the larvae of the Argasidae. Parasitology.

[CR49] Wágnerová M, Chalupková A, Hrabovská Z, Ančicová L, Mistríková J (2015). Possible role of different animal species in maintenance and spread of murine gammaherpesvirus 68 in the nature. Acta Virol.

